# Program Signaling in Emergency Medicine: The 2022–2023 Program Director Experience

**DOI:** 10.5811/westjem.19392

**Published:** 2024-08-27

**Authors:** Alexis E. Pelletier-Bui, Timothy Fallon, Liza Smith, Tania Strout, Michelle Fischer, Mark Olaf, Erin McDonough, Brian Barbas, Michael Cirone, Elizabeth Barrall Werley

**Affiliations:** * Cooper University Hospital/Cooper Medical School of Rowan University, Department of Emergency Medicine, Camden, New Jersey; † Maine Medical Center, Tufts University School of Medicine, Department of Emergency Medicine, Portland, Maine; ‡ University of Massachusetts Chan School of Medicine, Baystate Medical Center, Department of Emergency Medicine, Springfield, Massachusetts; § Penn State College of Medicine/Penn State Health Milton S. Hershey Medical Center, Department of Emergency Medicine, Hershey, Pennsylvania; ∥ Geisinger Commonwealth School of Medicine, Department of Emergency Medicine, Scranton, Pennsylvania; ¶ University of Cincinnati College of Medicine, Department of Emergency Medicine, Cincinnati, Ohio; # Loyola University Chicago, Stritch School of Medicine, Loyola University Medical Center, Department of Emergency Medicine, Maywood, Illinois; ** University of Illinois, Department of Emergency Medicine, Chicago, Illinois

## Abstract

**Introduction:**

Program signaling (PS), which enables residency applicants to signal their preference for a specific program, was introduced in emergency medicine (EM) in the 2022–2023 residency application cycle. In this study we evaluated EM program directors’ (PD) utilization of PS in application review and ranking. This study also explores the relationship between program characteristics and number of signals received as well as the relative importance and utilization of signals related to the number of signals received.

**Methods:**

This is an institutional review board-approved, cross-sectional study of PDs at Accreditation Council for Graduate Medical Education-accredited EM residency programs. We used descriptive statistics to describe the characteristics of residency programs and practices around PS. Measures of central tendency and dispersion summarized continuous variables. We used chi-square analysis or the Fisher exact test for comparisons between groups for categorical variables. Comparisons for continuous variables were made using the *t*-test for independent samples or analysis of variance.

**Results:**

The response rate was 41% (n = 113/277 EM programs). Most programs participated in PS (n = 261/277 EM programs, 94.2%). Mean number of signals received was 60 (range 2–203). Signals received varied based on program characteristics including geographic location and program type, duration, environment, and longevity. Most used PS in holistic review (52.2%), but other uses varied by proportion of applications that were signaled. The importance of PS in application review (mean 2.9; 1–5 scale, 1 = not important, 5 = extremely important) and rank list preparation (2.1) was relatively low compared to other application elements such as standardized letters of evaluation (4.97 for review, 4.90 for ranking).

**Conclusion:**

The study provides insights into PS utilization in EM’s inaugural year. We have identified patterns of signal use based on program characteristics and number of signals received that can inform signal allocation and utilization on an individual applicant and program level. A more nuanced understanding of signal use can provide valuable insight as the specialty of EM grapples with fluctuations in its applicant numbers and shifting demographics of its applicant pool.

Population Health Research Capsule What do we already know about this issue?

*Program signaling (PS) was introduced into the emergency medicine (EM) residency application process in 2022–2023 via the Electronic Residency Application Service.*

What was the research question?

*How did EM program directors use PS in application review and ranking?*

What was the major finding of the study?

*52.2% of program directors used PS in holistic review. Other uses varied by proportion of signaled applications.*

How does this improve population health?

*Understanding PS usage patterns helps inform PS allocation and usage on an individual applicant and program level.*

## INTRODUCTION

Program signaling (PS) was introduced into the residency application process in response to the increasing number of applications received by programs, exacerbating the challenge of comprehensive holistic review.^1^ Subsequently, EM has experienced drastic fluctuations in the number of applicants pursuing EM and specialty Match rates, as well as unprecedented changes to the demographics of its application pool over the last several years.^2^ Even with variability in the number of applications to emergency medicine (EM) in recent years, EM application numbers remain significantly above what they were 10 years ago.^2^ ^,^ ^3^ Program signaling allows applicants to assign signals to their most desired training programs, so that programs may focus their holistic efforts toward high-yield interview candidates, potentially benefiting both applicants and programs.

Program signaling was implemented in EM via the Electronic Residency Application Service (ERAS) in the 2022–2023 residency application cycle, allowing applicants to send five signals at the time of their residency application submission with instruction to not signal their home or away-rotation institutions.^4^ The Association of American Medical Colleges (AAMC) published generic guidance for programs regarding the use of PS only during the interview-offer phase and programs attested to a code of conduct regarding signal usage when opting into the process, including guidance not to use PS in rank order list (ROL) decisions.^5^ While data was evaluated by ERAS across all participating specialties, and other specialties have reported their own specialty-specific data, opportunities remained to further investigate questions specific to PS within EM.^6^ ^–^ ^18^ The unique challenges facing EM created an appetite and underscored the need for specialty-specific guidance.

To provide evidence-based guidance, the ERAS Application Working Group, a subset of the Council of Residency Directors in EM (CORD EM) Application Process Improvement Committee, created a survey to address more nuanced EM-specific questions not asked or answered by the AAMC survey. Our objective in this study was to determine how EM program directors (PD) used PS in their application review and ranking practices during the 2022–2023 application cycle, particularly in relation to the proportion of signaled applications received. To our knowledge, no other specialties participating in PS have reported PS utilization data in this manner. We also explored the relationship between program characteristics and the number of signals received, including characteristics not previously studied by the AAMC such as geographic location, program length of training, program environment, and program longevity. Lastly, we investigated the relative importance and utilization of signals in comparison to other residency application elements and in relation to the number of signals received.

## METHODS

### Study Design

We used a cross-sectional study design. Participants were PDs in Accreditation Council for Graduation Medical Education (ACGME)-accredited EM residency programs participating in the 2023 National Resident Matching Program Match. The CORD member directory, cross-referenced with the ACGME Accreditation Data System public search website, was used to compile the email distribution list. We edited the list to reflect new PDs when possible (277). The survey was created following a thorough literature review and synthesis of background information. Questions were iteratively reviewed by experts in EM medical education. The survey was further refined after conducting two cognitive interviews with EM residency program leaders and then piloted by several EM educators to assess for clarity of the questions. Data was primarily quantitative. No identifying information was collected. The study was designed to take about 10 minutes to complete. Our survey tool is included in Appendix 1. This study was approved by the institutional review board at the institution of authors TF and TS.

### Data Collection

The survey link was distributed via email. We collected data using a confidential and secure web-based (Qualtrics, Provo, UT) survey of EM residency PDs or their designees. Anonymous links were created for each potential respondent and distributed via Qualtrics. As described by Dillman and colleagues, one week prior to distribution of the survey link, PDs received a brief email introducing the study and informing them that they would receive the study link in the coming week.^19^ Participants then received a message containing the survey link. Non-responders received up to three reminder messages over five weeks.

### Data Analysis

Data was downloaded from REDCap, hosted at Maine Medical Center, directly into SPSS for Windows v 27 (IBM SPSS Statistics for Windows, IBM Corp., Armonk, NY) statistical software for analysis. We used descriptive statistics to describe the characteristics of study participants’ residency training programs. Program practices and experiences around PS were described using numbers and percentages for each categorical variable. We summarized continuous variables using measures of central tendency (mean or median) and dispersion (standard deviation, interquartile range [IQR]). Comparisons between groups for categorical variables were made using chi-square analysis or the Fisher exact test. Comparisons for continuous variables were made using the *t*-test for independent samples or analysis of variance. We accepted a *P*-value of <0.05 as significant. We also computed differences between groups and their associated 95% confidence intervals (CI) and created visual data displays to aid in interpretation.

## RESULTS

### Program Characteristics

We received 113/277 surveys (response rate 41%). Participants represented diverse geographic regions, with the largest numbers from the Middle Atlantic, East North Central Midwest, and South Atlantic regions (Table 1). Programs represented were most commonly urban, university-based, and three years length of residency training. Faculty at participating programs were largely university or hospital employees, and most programs reporting being founded more than 15 years.

**Table 1.** Characteristics of participating residency programs and survey respondents.

Characteristic	% (*n*)	Comparison to existing program data (percentage of programs)
Professional role		
^*^Program director	100 (113)	
Geographic region		
Middle Atlantic	24.8 (28)	23.7^a^
East North Central Midwest	20.4 (23)	20.5^a^
South Atlantic	17.7 (20)	19.1^a^
Pacific West	11.5 (13)	10.6^a^
West South Central	11.5 (13)	9.9^a^
New England	5.3 (6)	4.2^a^
Mountain West	4.4 (5)	3.9^a^
West North Central Midwest	2.7 (3)	3.9^a^
East South Central	1.8 (2)	4.2^a^
Program length		
Three years	77.0 (87)	80.6^b^
Four years	23.0 (26)	19.4^b^
Program environment		
Urban	63.7 (72)	Not available
Suburban	30.1 (34)	Not available
Rural	6.2 (7)	Not available
Program type		
University-based	47.8 (54)	35.4^a^
Community-based, university-affiliated	36.3 (41)	46.2^a^
Community-based	15.9 (18)	18.4^a^
Faculty employment model		
University or hospital	73.5 (83)	Not available
Contract management group	18.6 (21)	Not available
Democratic physician-led group	8.0 (9)	Not available
Program longevity		
<5 years	17.7 (20)	Not available
5–10 years	8.0 (9)	Not available
10–15 years	10.6 (12)	Not available
>15 years	63.7 (72)	Not available

* *261/277 EM programs participated in PS for 2022–2023. All 277 programs surveyed.*

Middle Atlantic = NJ, NY, PA; East North Central Midwest = IL, IN, MI, OH, WI; South Atlantic = DC, DE, GA, FL, MD, NC, SC, VA, WV, PR; Pacific West = AK, CA, HI, OR, WA; West South Central = AR, LA, OK, TX; New England = CT, MA, ME, NH, RI, VT; Mountain West = AZ, CO, ID, MT, NM, NV, UT, WY; West North Central Midwest = IA, KS, MN, MO, ND, NE, SD; East South Central = AL, MS, KY, TN.

a Fellowship and Residency Electronic Interactive Database (FREIDA), https://freida.ama-assn.org

b Emergency Medicine Residents’ Association (EMRA) Match Database, https://match.emra.org/

### Program Signaling Participation and Applications Received

The majority of respondents participated in the PS component of the ERAS supplemental application during the 2022–2023 residency application cycle (106, 94%). Reasons for non-participation included not signing up in time (three, 2.7%), feeling that it would not contribute to applicant review or interview offer decisions (two, 1.8%), and being a newly approved program (1, 0.9%). Programs interviewed to fill a mean and median of 12 postgraduate year (PGY)-1 spots (range 6–26 spots, IQR 8–15). The number of signals received by participating programs ranged from 2–203, with a mean of 60 and median of 50 (IQR 23–86). Programs reported receipt of between 283–1,400 applications (mean 768, median 772, IQR 600–926). The proportion of applications that were signaled ranged from 0.7% to 26.5% (mean 7.3%, median 6.5%, IQR 3.9–10.1%).

There was a moderate, positive correlation between the number of signals and the number of applications received (*r* = 0.581, *P* < 0.001) and the proportion of signals received increased based upon the number of applications received (*P* < 0.001) as well as the proportion of applications that were signaled (*P* < 0.001). The number of signals received increased as the number of PGY-1 positions increased (*P* < 0.001). Four quartiles were determined for the number of program signals received, the number of applications received, and the proportion of applications signaled (Supplemental Table 1) to allow for further comparison of data as subsequently detailed.

### Signals Received by Program Characteristics

**Figure 1.**
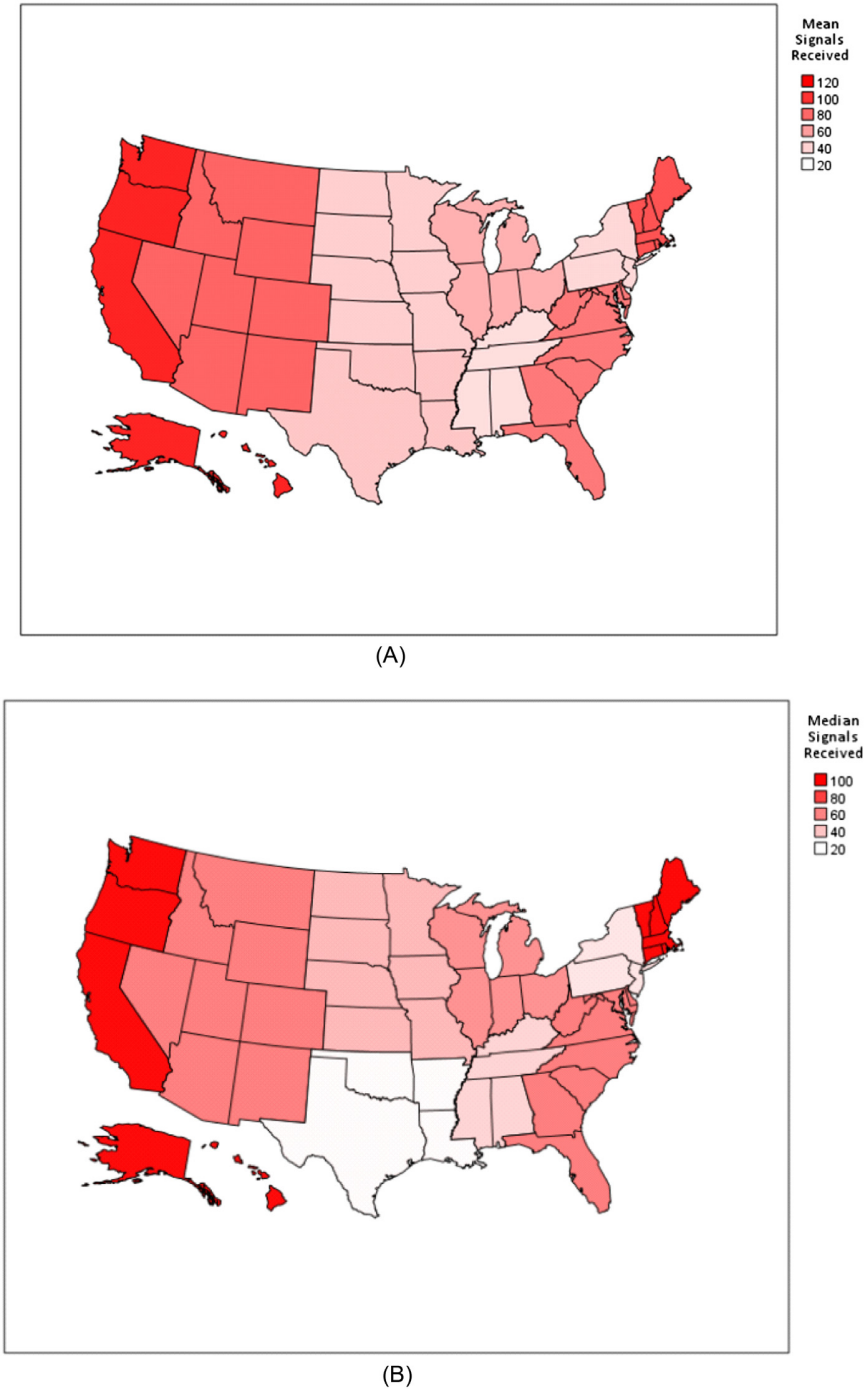
(A) Mean number of signals received by geographic region. (B) Median number of signals received by geographic region. Geographic regions include: East North Central Midwest (IL, IN, MI, OH, WI), East South Central (AL, MS, KY, TN), Middle Atlantic (NJ, NY, PA), Mountain West (AZ, CO, ID, MT, NM, NV, UT, WY), New England (CT, MA, ME, NH, RI, VT), Pacific West (AK, CA, HI, OR, WA), South Atlantic (DC, DE, GA, FL, MD, NC, SC, VA, WV, PR), West North Central Midwest (IA, KS, MN, MO, ND, NE, SD), and West South Central (AR, LA, OK, TX).

**Figure 2.**
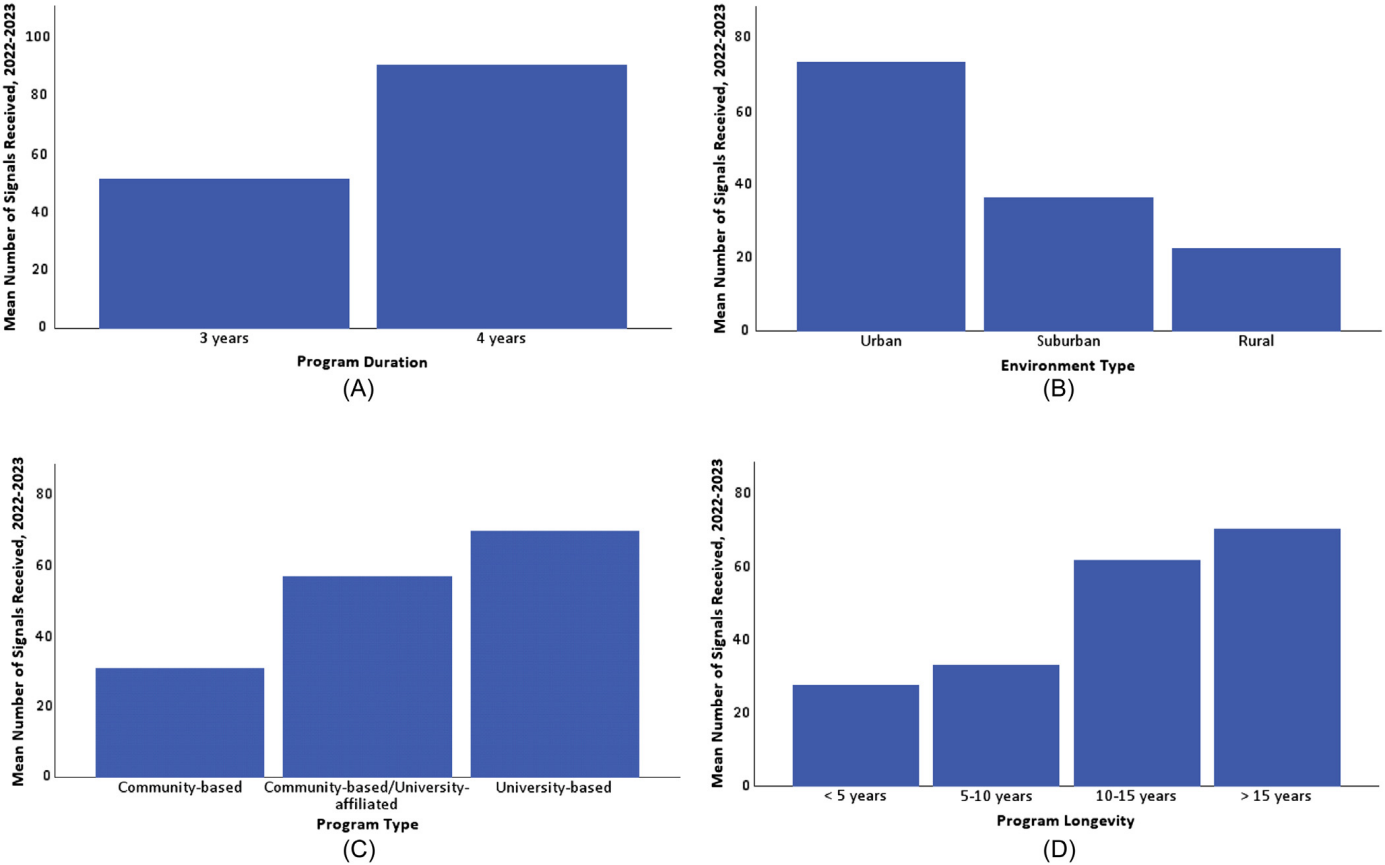
Mean number of signals received by program characteristics. (A) Mean number of signals received by program duration. (B) Mean number of signals received by environment type. (C) Mean number of signals received by program type. (D) Mean number of signals received by program longevity.

The number of signals received differed significantly based on several key characteristics: geographic location, with greater numbers of signals received in coastal regions (*P* < 0.01); program duration, with four-year receiving more than three-year programs (*P* < 0.01); program type, with urban programs receiving the most (*P* < 0.01); program environment, with university-based programs receiving the most (*P* < 0.01); and longevity of programs with programs in existence >15 years receiving the most (*P* < 0.01). Additional detail is provided in Figures 1 and 2 and Supplemental Figure 1.

### Signal Utilization

**Figure 3.**
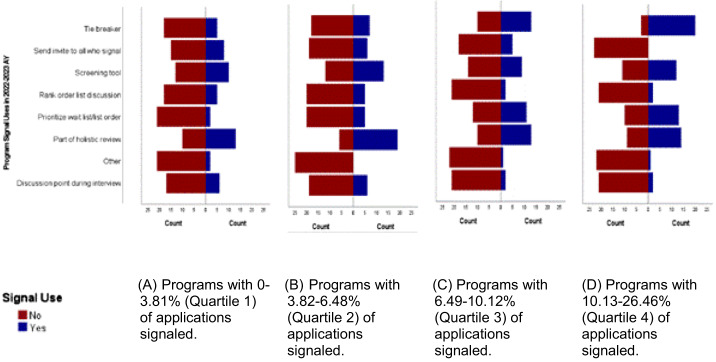
Program signal use in the 2022–2023 academic year by the proportion of applicants signaled.^*^ ^*^The AAMC Code of Conduct, which programs attest to when signing up to participate in program signaling (PS), specifically prohibits the use of PS in rank-order list discussion and preparation.

Programs most commonly endorsed using PS as one component of holistic review (59, 52.2%). Additional specific ways that signals were used include the following: as a tiebreaker between two equally qualified candidates (45, 39.8%); as a screening tool (44, 38.9%); to help prioritize the program’s wait list or wait list order (31, 27.4%); and to send an interview invitation to every applicant who signaled the program (19, 16.8%). The proportion of applications that were signaled appeared to affect the frequency with which programs endorsed using signals to prioritize the wait list (*P* < 0.001), serve as a tiebreaker (*P* < 0.001), and to send interview invitations to every signaling applicant (*P* = 0.03) (Figure 3). Participants anticipated using PS in the 2023–2024 cycle similarly to their reported use in the 2022–2023 cycle, and similar differences were also noted for anticipated use based on the proportion of applications that were signaled.

### Signal Importance

Participants rated the importance of various application elements when considering interview invitations and preparing their program’s rank order list (ROL) using a 5-point scale (1 = not important at all, 5 = extremely important) (Table 2). Participants rated the standardized letter of evaluation (SLOE) as the most important element when reviewing applications (mean 4.97, 95% CI 4.93–5.00). The SLOEs (mean 4.90, 95% CI 4.83–4.97) and interview day performance (mean 4.81, 95% CI 4.72–4.89) were most important when preparing the ROL. Importance of the presence or absence of a program signal when reviewing applications was a mean of 2.9 (95% CI 2.67–3.13) and median of 3 (2,4). Importance of the presence or absence of a program signal when preparing a ROL was a mean of 2.1 (95% CI 1.87–2.32) and median of 2 (1–3). About 30% of participants (28) endorsed the presence or absence of a program signal as very or extremely important when reviewing applications while 11% (10) rated program signals as being equally important to ROL development.

**Table 2.** Importance of application elements.

Application element	Importance when reviewing applications	Importance when preparing rank order list
	Mean (95% CI)	Mean (95% CI)
SLOEs	4.97 (4.93–5.00)	4.90 (4.83–4.97)
Interview day interactions	N/A	4.81 (4.72–4.89)
Prior work or life experiences	3.61 (3.42–3.80)	3.52 (3.32–3.72)
Board scores	3.47 (3.27–3.66)	3.14 (2.93–3.35)
MSPE	3.44 (3.24–3.65)	3.32 (3.12–3.53)
Extracurricular involvement	3.36 (3.17–3.54)	3.25 (3.05–3.45)
Presence or absence of a program signal	2.90 (2.67–3.13)	2.10 (1.87–2.32)
Communication before interview	2.64 (2.42–2.87)	2.89 (2.65–3.13)
Research experience	2.46 (2.27–2.64)	2.43 (2.24–2.62)
Letters of recommendation	2.40 (2.22–2.58)	2.33 (2.15–2.52)

* 5 point scale where 5 = extremely important and 1 = not important at all.

*CI*, confidence interval; *SLOE*, standardized letter of evaluation; *MSPE*, medical student performance evaluation.

We assessed for differences in PDs’ relative assessments of various application elements based on the proportion of applications that were signaled (Supplemental Figure 2). As the proportion of applications signaled increased, the proportion of programs endorsing board scores as “extremely important” decreased (*P* < 0.01). As the proportion of applications signaled increased, the proportion of programs endorsing communication before the interview as “not important at all” increased while the proportion rating this factor “very important” decreased (*P* < 0.01). Extracurricular involvement increased in importance as the number of applications signaled increased, with a larger proportion of participants rating this aspect of the application “extremely important” as the proportion of applications signaled increased (*P* = 0.04). Programs with the lowest proportion of signaling applicants were more likely to rate research experience as “not important at all” than those who had a larger proportion of applications signaled (*P* = 0.02).

## DISCUSSION

Responses to our survey appear to be appropriately representative of programs nationwide with regard to geographic distribution, program length, and program type (Table 1).^20^ ^,^ ^21^ Ranges and median numbers for applications and PS data are similar to ERAS data, again demonstrating that our survey respondents reflected a representative sample of EM programs that participated in PS during the studied application cycle.^6^

For data analysis, we used quartiles based on the percentage of signaling applications a program received to correct for the differences in raw numbers based on program size. With the number of signals allocated to each EM applicant increasing from five to seven for the 2023–2024 academic year, it is reasonable to presume that the raw number and percentage of signaling applicants programs receive will also proportionally increase. This discrepancy may make it more difficult for a program to accurately identify with a given quartile based on this year’s application data, but these data should still serve as a rough guide by which programs can assess themselves.

Understanding the relationship between program characteristics and the number of received program signals can be helpful for both programs and applicants. Programs can determine their competitiveness within the context of similar programs, which can be particularly helpful in the current EM match environment with a changing applicant demographic pool and many programs going unmatched over the past few years.^2^ Providing programs with a barometer against which to measure their own demographics and proportion of signaled applicants early in the application cycle can help guide how they incorporate program signals into their approach and more effectively select applicants who will be highest yield for their programs. By understanding signaling trends as related to program characteristics, advisors and applicants may be able to strategically determine the best approach for allocating signals to maximize each signal’s impact.

In our study, we noted that the Pacific West and New England regions demonstrated the highest mean and median signal numbers. In contrast, programs in the East South Central, Mid-Atlantic, West South Central, and West North Central Midwest received fewer signals. It is reasonable to speculate that many of these patterns reflect overall population density patterns, suggesting local preferences that mirror the US population. This hypothesis aligns with our data, which showed that more urban (likely more population-dense) programs received a higher proportion of signals. The only region that does not fit this hypothesis is the Mid-Atlantic region, which is the most densely populated in the country, but we suspect the very high EM program density in this region likely contributed to program signal dilution, leading to lower signals per program.

On average, four-year programs received a higher proportion of signaling applicants than three-year programs. While program length itself may be a driver of this, it may also be due to other confounding features more commonly associated with four-year programs, including urban location, university affiliation, and program duration and stability. Ultimately, our data was unable to discern this difference. Programs with the lowest proportion of signaling applicants were more likely to be smaller, rural, and not academically affiliated. These programs were more likely to rate research experience as “not important at all.” We suspect that these smaller, more community-oriented programs may be less research-focused in their missions and, therefore, emphasize research less in their applicant selection. Applicants may be able to use this information to target their signals depending on their interests.

It seems intuitive that the proportion of signaling applicants a program receives would affect how that program values and uses the signal, but to our knowledge this is the first data to demonstrate that effect. When examining signaling use among programs separated into quartiles based on the proportion of signaling applicants, significant differences emerged. Programs that received lower proportions of signaling applicants were more likely to report offering interviews to all signaling applicants while those with the highest proportion of signaling applicants were more likely to incorporate signals as a screening tool or to help prioritize the program’s wait list or wait-list order.

By asking programs to rate the importance of various application elements, we hoped to gain an understanding of the relative importance of PS in relation to interview offers and ROL creation. Receiving a program signal in orthopedics was ranked among the most important factors in resident selection for interview.^13^ While a successful sub-internship at the PD’s institution and letters of recommendation were the highest-ranked criteria for resident selection for interview at urology programs, 81% of urology PDs reported that a lack of a signal would negatively impact interview offer chances for an applicant.^18^ In our study, program signals were not shown to hold as much weight as in orthopedics or urology. Program signals were only rated as more important than narrative letters of recommendation, pre-interview communication, and research experience.

How an applicant performs clinically (SLOEs, Medical Student Performance Evaluation) is understandably most important, with PS intended to be only one small part of the holistic application review.^22^ Students can be reassured that the traditionally valued portions of the EM application retain their importance well above the value of a program signal, and programs across all quartiles are interviewing and ranking students who did not send them a signal.

Analyzing this data in a more granular fashion, we did observe some significant differences in the relative importance of residency application elements between quartiles. As the proportion of signaling applicants increased, the proportion of participants endorsing board scores as “extremely important” decreased. This discrepancy may speak to the intended ability of PS to mitigate the use of filtering behavior. Programs with smaller proportions of signaling applicants may continue to seek out strategies to stratify their applicant pool to better allocate their holistic review efforts, such as using board score filters. Programs with a higher proportion of signaling applicants, on the other hand, may not feel this same pressure. Alternatively, it is possible that having been prompted by the introduction of PS to investigate programs before applying, applicants may strategically have chosen to target their signals to programs that advertised a lack of board score cutoffs because their score fell below stated cutoffs at other programs or because they valued programs that do not emphasize standardized test scores.

Our data also demonstrates that as the proportion of signaling applicants increased, the proportion of respondents rating pre-interview communication as “extremely important” decreased and the proportion of respondents rating pre-interview communication as “not important at all” increased. This trend suggests that the signal is serving its intended purpose of allowing the applicant to meaningfully express interest, obviating the need for additional, extra-application communication, lessening the burden for both applicants and programs. It also suggests that PS reduces the impact of other communication from applicants.

The AAMC guidance was consistent in its messaging that program signals were only to be used during the application review and interview-offer portion of the application cycle. It is worth noting that despite all programs having attested in the code of conduct not to use PS in the consideration of ROL placement, 11% of programs reported program signals to be very important to the ROL development process. The 2022–23 AAMC PD survey found similar results among PD respondents from all specialties.^6^ Program directors may be extrapolating that a student who signaled is likely to be a higher probability match than a student who did not send a signal. This use presumes that student preference will not be significantly affected by their experiences engaging with programs throughout the interview season and is at risk of being flawed logic. However, it is important that applicants be aware that signals may be used by PDs in this manner and should take this into consideration when choosing where to signal.

Participation of EM programs in PS remained robust for the 2023–2024 cycle, with 278 of 279 programs participating and 97.5% of applicants participating (email communication from AAMC ERAS Pilot Administration Director, Jayme Bograd, January 2024).^24^ We hope that this data helps inform programs and applicants on a more nuanced approach to PS in the EM residency application process.

## LIMITATIONS

Respondents (113) compared to the total number of ACGME-accredited EM residency programs (277) was limited. The PDs who chose to respond may differ from those who did not concerning their PS experience. Forty-six percent of EM programs did not fill in the 2023 Main Residency Match.^25^ Our survey was distributed in the weeks that followed. The PDs experiencing a difficult Match cycle may have been more or less inclined to fill out a survey regarding the residency application process. University-based programs were over-represented. Community-based, university-affiliated programs were under-represented. The 11% of programs that reported using signals as part of their ROL discussions may be an underestimate as other programs may not have been comfortable disclosing behavior that was knowingly in violation of the code of conduct.

## CONCLUSION

This study provides detailed data and patterns of signal use yielding insights into program signaling in EM’s inaugural year for both programs and applicants. Our data provides a more nuanced understanding of signal utilization across a spectrum of EM programs in a way that allows individual programs to go beyond the general AAMC recommendations and compare their approach to that of programs with similar characteristics. Identifying patterns of signal use based on program characteristics can also inform advising for students deciding on how to best allocate their signals. As EM continues to navigate fluctuations in its applicant numbers and shifting demographics of its applicant pool, providing insight to guide signal use and utilization can help pave a path forward for the specialty toward the goal of more efficiently finding the right applicant for the right program.

## Supplementary Information









