# Supplementary material for: Program Signaling in Emergency Medicine: The 2022–2023 Program Director Experience
Source: West J Emerg Med. 2024 Aug 27;25(5):715–24. doi: 10.5811/westjem.19392 (PMC11418878; doi:10.5811/westjem.19392)
Supplement: Supplementary file 1 [file wjem-25-715-s001.docx]

On behalf of the CORD Application Process Improvement Committee Program Signaling Working Group, we invite you to participate in a brief on-line survey which is designed to understand Emergency Medicine Program Directors’ thoughts and practices regarding the use of Program Signaling during the 2022-2023 residency application cycle. Results of this survey will be published and utilized to help inform the future direction of program signaling within emergency medicine. This survey should not take you more than 10 minutes to complete. Participation in this study is voluntary and you may withdraw at any time. No individual identifying information will be collected or maintained. This study has been reviewed by the X IRB and given exempt status. Any questions or concerns may be directed to X at X. Clicking to proceed will serve as your consent to participate in the study.

**PART 1: Demographic Information**

1. Are you an Emergency Medicine Program Director who participated in the 2022-2023 interview season? (If you are completing on behalf of the PD, please select Yes)

- Yes > proceed
- No > submit survey
  - You selected “No” when asked if your are an Emergency Medicine Program Director (or completing on behalf of the PD). By selecting no, you will be taken to the end of the survey and will not have an opportunity to answer any other questions. If this is correct, please confirm below and enter. If this was an incorrect response, please use the back button to edit your response now.

1. The geographic region of my residency program is:

- East North Central Midwest (IL, IN, MI, OH, WI)
- East South Central (AL, MS, KY, TN)
- Middle Atlantic (NJ, NY, PA)
- Mountain West (AZ, CO, ID, MT, NM, NV, UT, WY)
- New England (CT, MA, ME, NH, RI, VT)
- Pacific West (AK, CA, HI, OR, WA)
- South Atlantic (DC, DE, GA, FL, MD, NC, SC, VA, WV, PR)
- West North Central Midwest (IA, KS, MN, MO, ND, NE, SD)
- West South Central (AR, LA, OK, TX)

1. The length of my residency program is:
   - 3 years
   - 4 years
2. My program type is best defined as:
   - Community-based
   - Community-based / University-affiliated
   - University based
3. My **primary training site** program environment is:
   - Urban
   - Suburban
   - Rural
4. Faculty at my program are employed by:
   - University / Hospital
   - Contract management group (i.e. Team Health, US Acute Care Solutions, etc)
   - Democratic physician-led group
5. My residency program has been in existence for:
   - <5 years
   - 5-10 years
   - 10-15 years
   - >15 years

**PART 2: PROGRAM SIGNALING PARTICIPATION**

1. Did you participate in the Program Signaling component of the ERAS Supplemental Application during the 2022-2023 residency application cycle?
   - Yes > proceed to question PART 3
   - No > proceed to next question
2. Why did you not participate in the Program Signaling component of the ERAS Supplemental Application during the 2022-2023 residency application cycle? (choose all that apply) > SUBMIT SURVEY AFTER QUESTION COMPLETED
   - Did not sign up in time
   - Did not feel it would contribute to applicant file review / interview offer decisions
   - Other (free text)

**PART 3: PROGRAM SIGNALING EXPERIENCE**

10. How many signals did your program receive for the 2022-2023 residency application cycle? (raw number)

11. How many applications did your program receive for the 2022-2023 residency application cycle? (raw number)

12. How many PGY1 positions were you interviewing for? (raw number)

13. How did you utilize program signals during the application process during the 2022-2023 residency application season? (choose all that apply)

- As a screening tool
- Send an interview to every applicant who signaled my program
- Tie breaker between two equally qualified applicants
- Prioritizing wait list / wait list order
- Just one part of a holistic review
- Discussion point during interview (example: asking an applicant why they signaled you)
- During rank order list discussion
- Other (free text response)

14. How do you anticipate using signals during the UPCOMING 2023-2024 residency application season? (choose all that apply)

- As a screening tool
- Send an interview to every applicant who signaled my program
- Tie breaker between two equally qualified applicants
- Prioritizing wait list / wait list order
- Just one part of a holistic review
- Discussion point during interview (example: asking an applicant why they signaled you)
- During rank order list discussion
- Other (free text response)

15. Did signals save you time during file review?

- Yes
- No
- Unsure

16. Did you allow all interviewing faculty to see whether or not an applicant signaled?

- Yes > branch to Q19
- No > branch to Q18

17. Which members of the interviewing faculty were aware of whether or not an applicant signaled? (choose all that apply) > branch to 18

- Program Director (PD)
- Assistant/Associate PD
- Clerkship Director/Assistant Clerkship Director
- Chair/Vice Chair
- Other (free text response)

18. How did you blind interviewers to the presence of a program signal?

- Free text response

19. With 5 being extremely important and 1 being not at all important, please rate how important the following elements of the application were to you/your program when reviewing applications/offering interviews:

Likert scale for each element:

Standardized Letters of Evaluation (SLOEs)

Other LORs

MSPE

Board Scores (USMLE and/or COMLEX)

Presence/Absence of a Program Signal

Research Experience

Prior work/life experience

Extracurricular involvement

Communications before interview with anyone associated with program (coordinator, chiefs, etc.)

20. With 5 being extremely important and 1 being not at all important, please rate how important the following elements of the application were to you/your program when preparing your program's rank order list:

Likert scale for each element:

Standardized Letters of Evaluation (SLOEs)

Other LORs

MSPE

Board Scores (USMLE and/or COMLEX)

Presence/Absence of a Program Signal

Research Experience

Previous work/life experience

Extracurricular involvement

Communications before/after interview with anyone associated with program (coordinator, chiefs, etc.)

Interview Day Interactions

21. Which students or groups of students did you perceive were unduly negatively impacted by the use of program signaling? [option to free text why they felt that way for each one chosen]

- None [with hard stop to not allow others to be chosen]
- Couples match applicants
- Orphan applicants (applicants without a home EM residency program)
- Applicants late to EM
- IMG applicants
- DO applicants
- Military match applicants
- Re-applicants to emergency medicine
- Underrepresented in medicine (URiM)
- Other (free text)

22. As a PD, did you find anything confusing or unclear about PS messaging?

- No > branch to next question
- Yes > branch to question below
  - What did you feel was confusing or unclear about PS messaging?

23. Were there any other unanticipated consequences or results of program signaling for your individual program?
