# Supplementary material for: Program Signaling in Emergency Medicine: The 2022–2023 Program Director Experience
Source: West J Emerg Med. 2024 Aug 27;25(5):715–24. doi: 10.5811/westjem.19392 (PMC11418878; doi:10.5811/westjem.19392)
Supplement: Supplementary file 2 [file wjem-25-715-s002.docx]

Supplemental Figure 1. Mean number of signals received by program characteristics and geographic region.


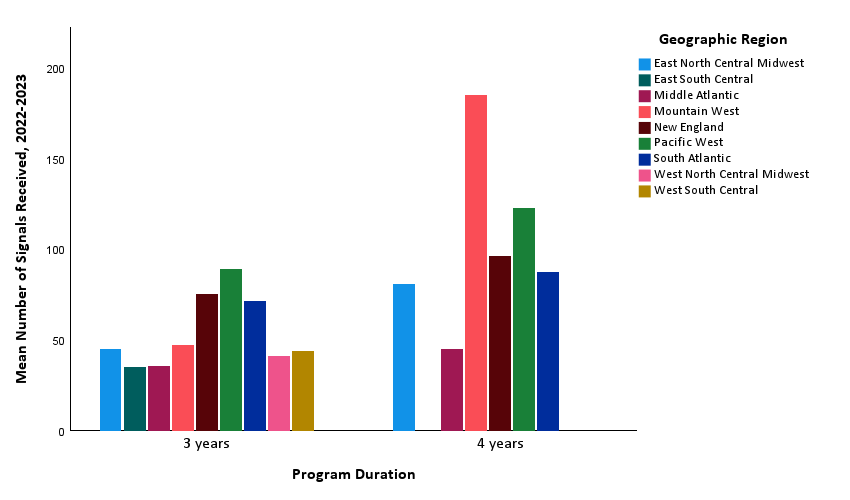


Panel A. Mean number of signals received by program duration.


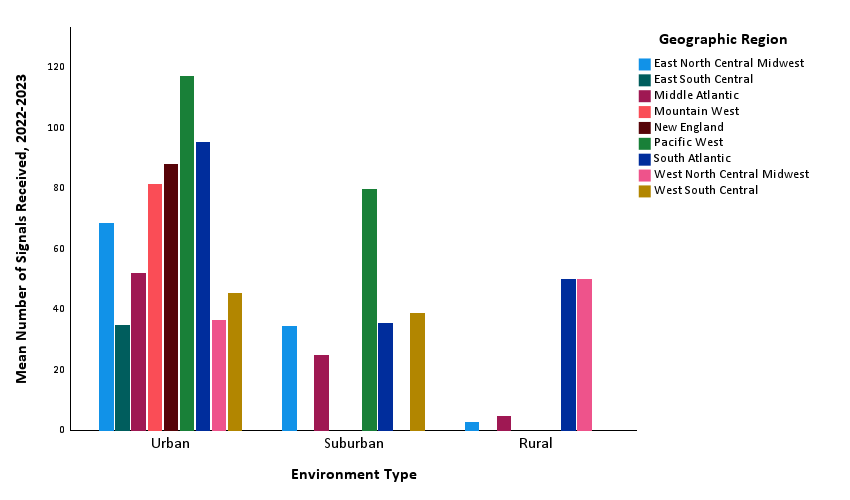


Panel B. Mean number of signals received by environment type.


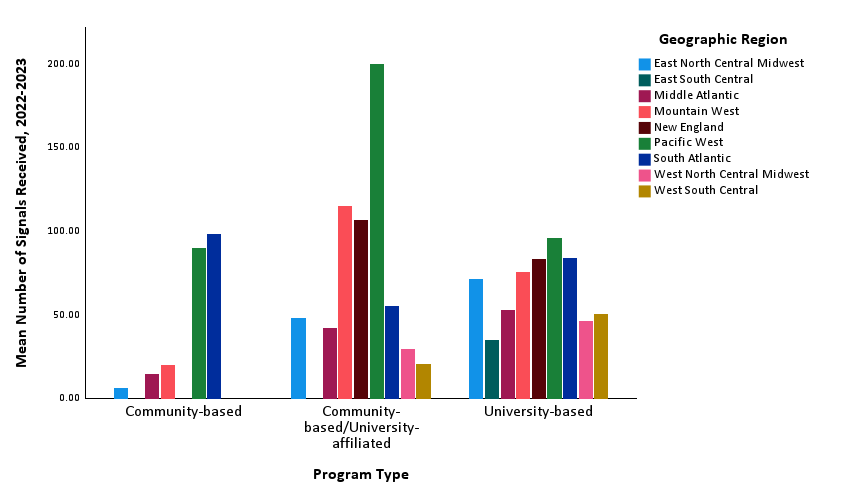


Panel C. Mean number of signals received by program type.


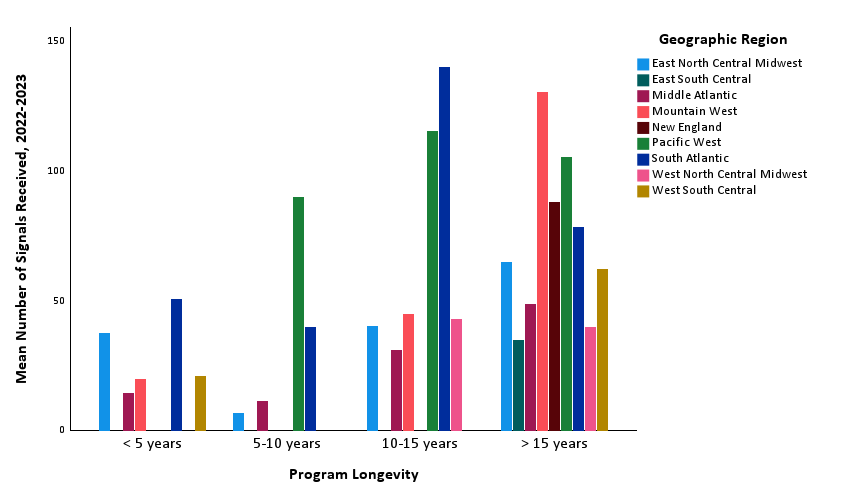


Panel D. Mean number of signals received by program longevity.
