# Supplementary material for: Program Signaling in Emergency Medicine: The 2022–2023 Program Director Experience
Source: West J Emerg Med. 2024 Aug 27;25(5):715–24. doi: 10.5811/westjem.19392 (PMC11418878; doi:10.5811/westjem.19392)
Supplement: Supplementary file 3 [file wjem-25-715-s003.docx]

Supplemental Figure 2. Anticipated program signal use in the 2023-2024 academic year by the proportion of applications signaled.


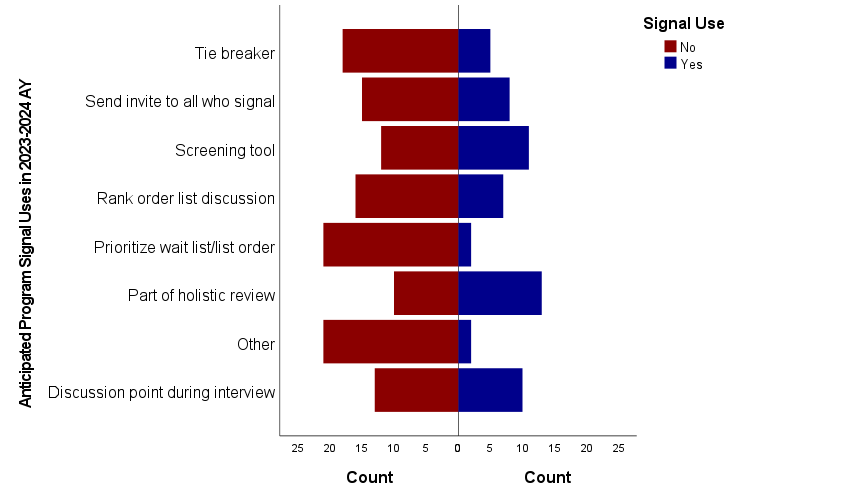


Panel A. Anticipated program signal use in the 2023-2024 academic year for programs with 0 – 3.81% (Quartile 1) of applications signaled.


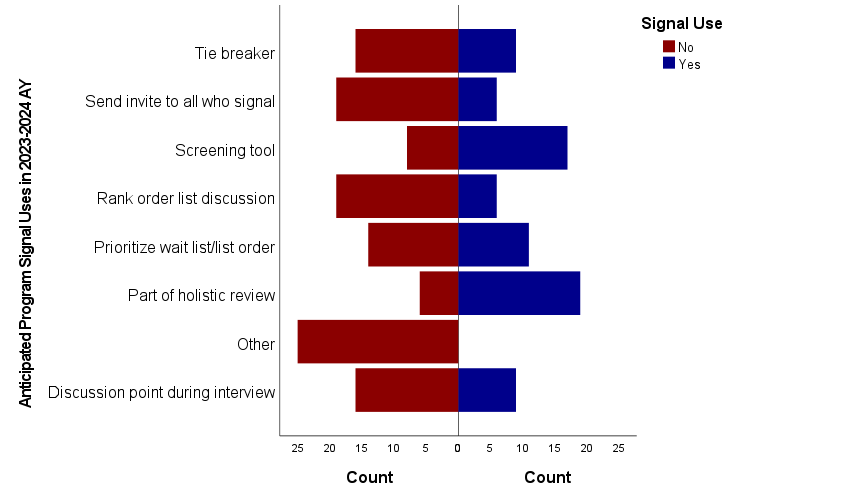


Panel B. Anticipated program signal use in the 2023-2024 academic year for programs with 3.82 – 6.48% (Quartile 2) of applications signaled.


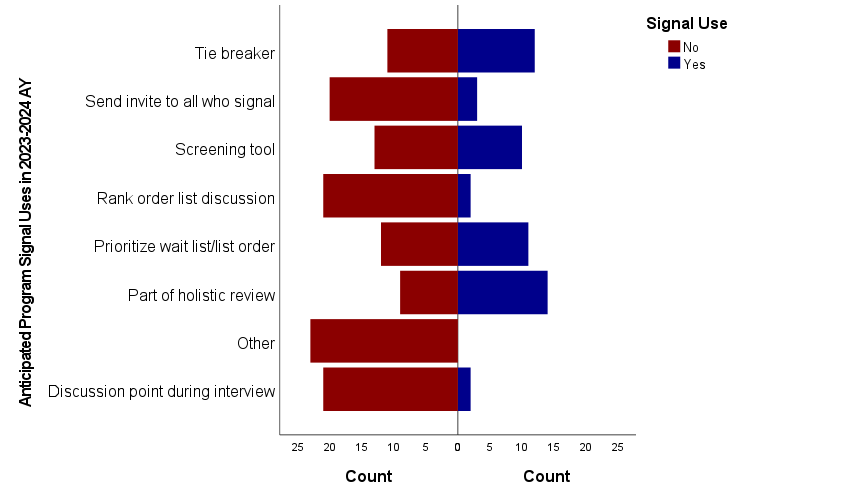


Panel C. Anticipated program signal use in the 2023-2024 academic year for programs with 6.49 – 10.12% (Quartile 3) of applications signaled.


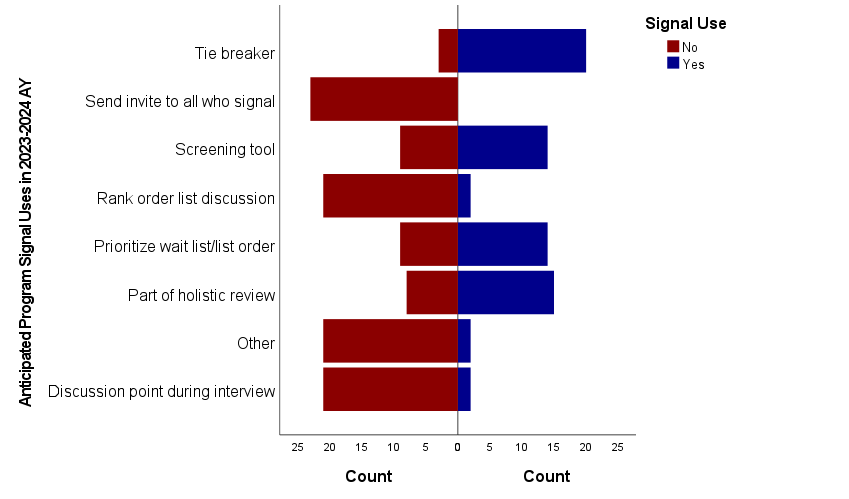


Panel D. Anticipated program signal use in the 2023-2024 academic year for programs with 10.13 – 26.46% (Quartile 4) of applications signaled.
