# Supplementary material for: Program Signaling in Emergency Medicine: The 2022–2023 Program Director Experience
Source: West J Emerg Med. 2024 Aug 27;25(5):715–24. doi: 10.5811/westjem.19392 (PMC11418878; doi:10.5811/westjem.19392)
Supplement: Supplementary file 4 [file wjem-25-715-s004.docx]

Supplemental Figure 3. Application element importance when reviewing applications by proportion of applications signaled (quartiles).


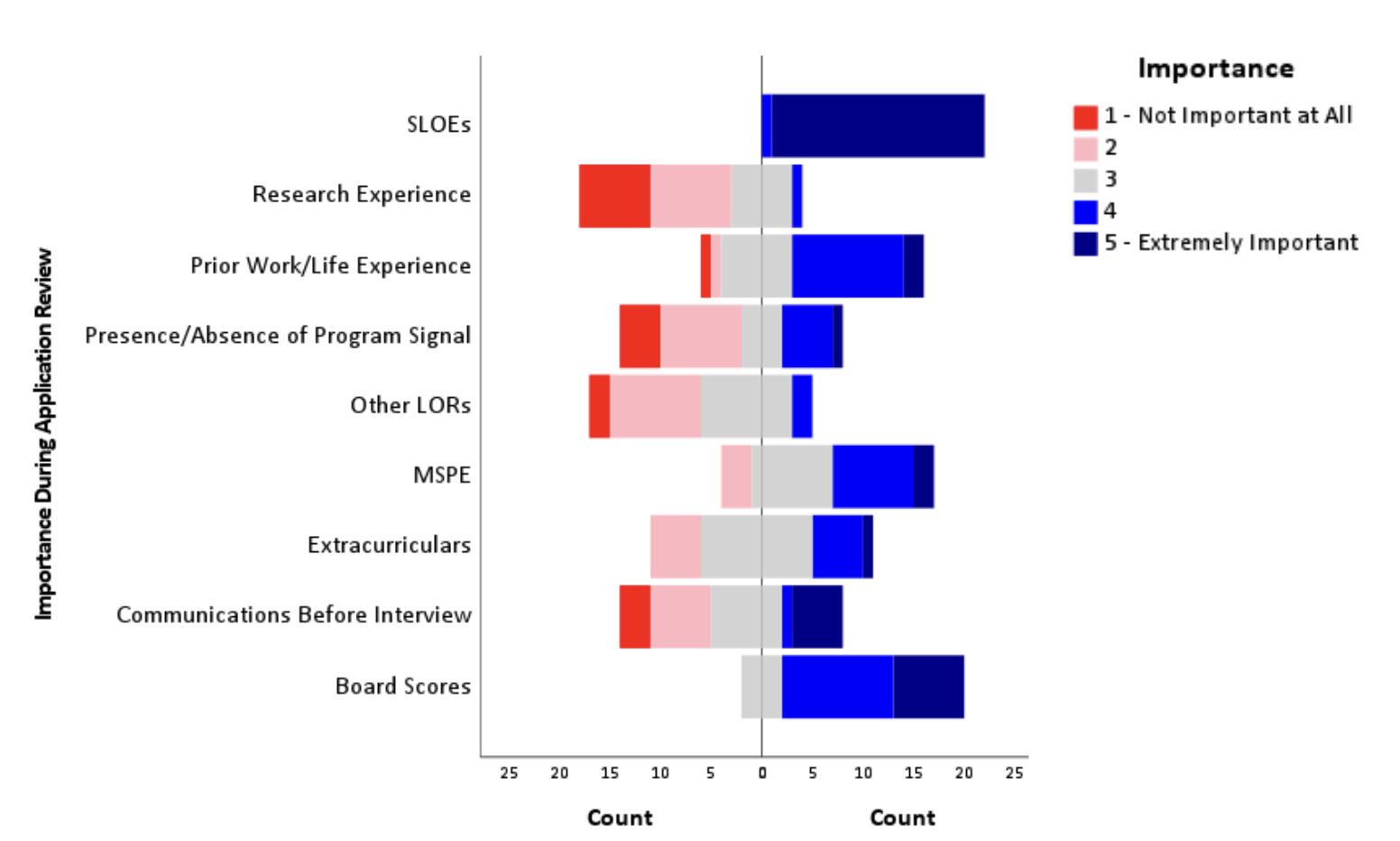


Panel A. Application element importance when reviewing applications by proportion of applications signaled (Quartile 1: 0 – 3.81% of applications signaled).


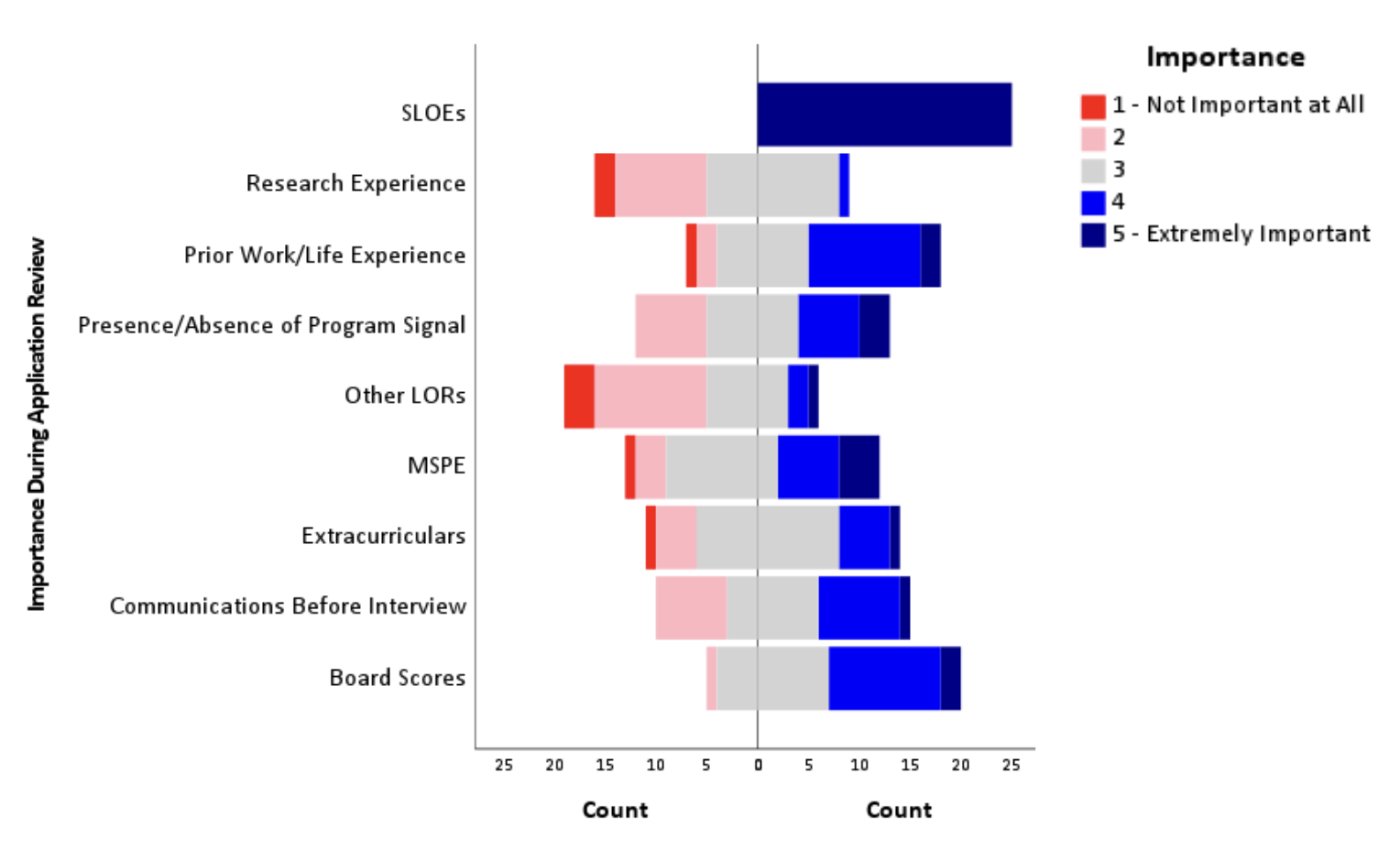


Panel B. Application element importance when reviewing applications by proportion of applications signaled (Quartile 2: 3.82 – 6.48% of applications signaled).


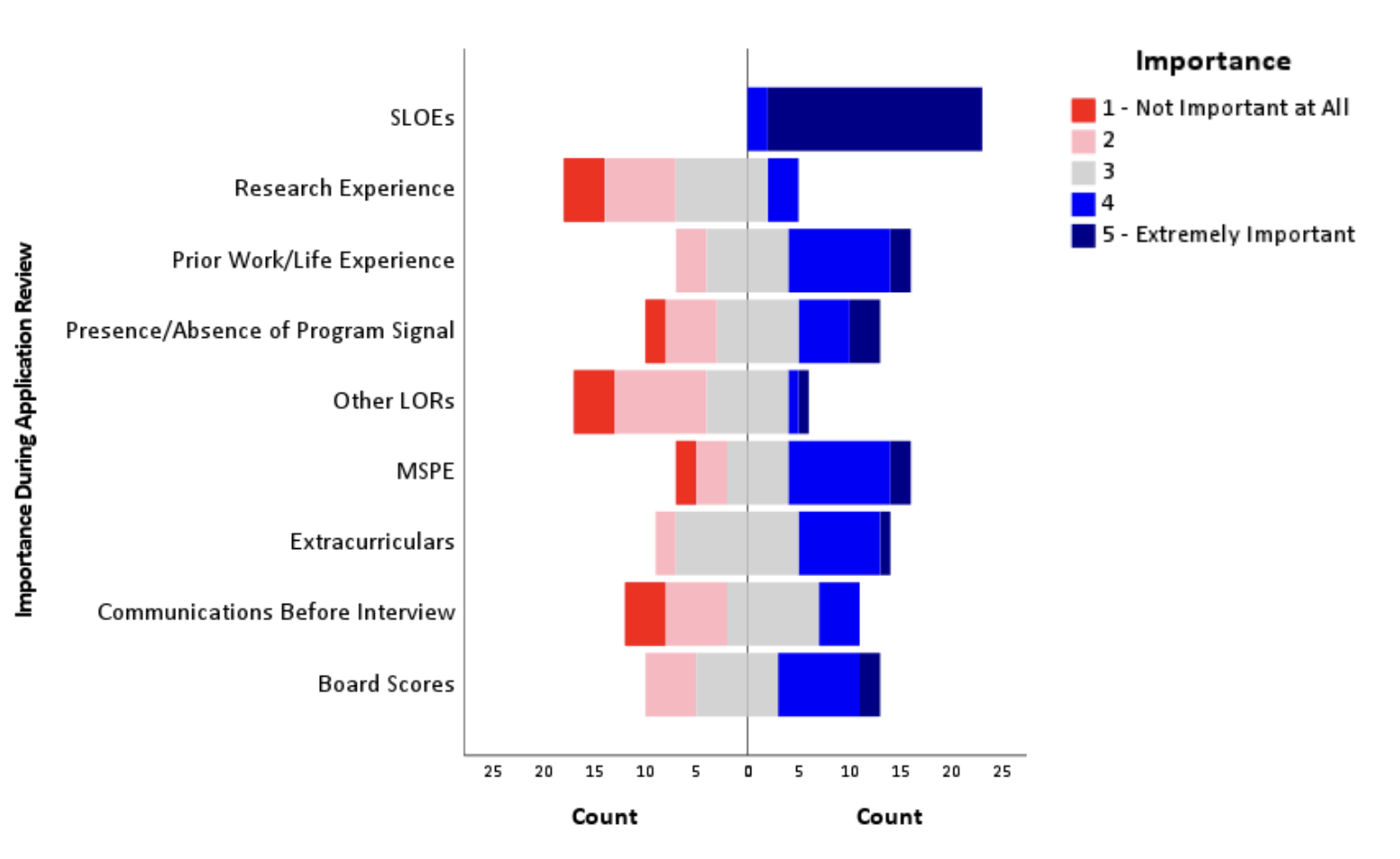


Panel C. Application element importance when reviewing applications by proportion of applications signaled (Quartile 3: 6.49 – 10.12% of applications signaled).


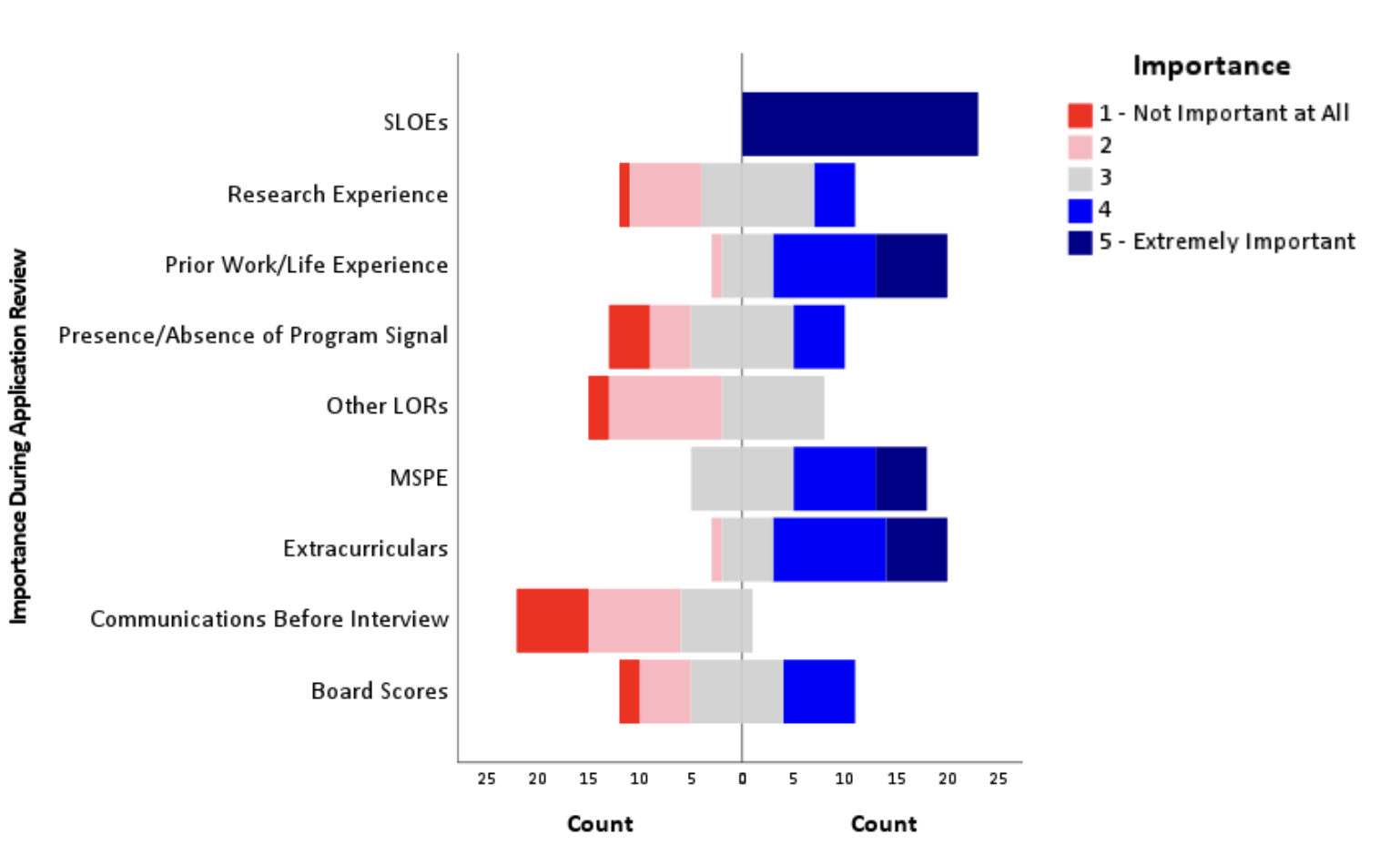


Panel D. Application element importance when reviewing applications by proportion of applications signaled (Quartile 4: 10.13 – 26.46% of applications signaled).
