# Supplementary material for: Program Signaling in Emergency Medicine: The 2022–2023 Program Director Experience
Source: West J Emerg Med. 2024 Aug 27;25(5):715–24. doi: 10.5811/westjem.19392 (PMC11418878; doi:10.5811/westjem.19392)
Supplement: Supplementary file 5 [file wjem-25-715-s005.docx]

Supplemental Table 1. Program signals and applications received subdivided into quartiles.

| Program Signals Received | *n* (%) |
| --- | --- |
| Quartile 1 (0-23 signals) | 24 (25.5) |
| Quartile 2 (24-50 signals) | 27 (28.7) |
| Quartile 3 (51-86 signals) | 21 (22.3) |
| Quartile 4 (87-203 signals) | 22 (23.4) |
|  |  |
| Applications Received |  |
| Quartile 1 (0-576 applications) | 22 (23.4) |
| Quartile 2 (577-760 applications) | 24 (25.5) |
| Quartile 3 (761-921 applications) | 24 (25.5) |
| Quartile 4 (922-1400 applications) | 24 (25.5) |
|  |  |
| Proportion of Applications Signaled |  |
| Quartile 1 (0-3.81% of applications signaled) | 23 (24.5) |
| Quartile 2 (3.82-6.48% of applications signaled) | 25 (26.6) |
| Quartile 3 (6.49-10.12% of applications signaled) | 23 (24.5) |
| Quartile 4 (10.13-23.46% of applications signaled) | 23 (24.5) |
