# Supplementary material for: Program Signaling in Emergency Medicine: The 2022–2023 Program Director Experience
Source: West J Emerg Med. 2024 Aug 27;25(5):715–24. doi: 10.5811/westjem.19392 (PMC11418878; doi:10.5811/westjem.19392)
Supplement: Supplementary file 6 [file wjem-25-715-s006.docx]

Supplemental Table 2. Program signal use and anticipated use.

| Program Signal Use | 2022-2023  *n* (%) | Anticipated 2023-2024  *n* (%) |
| --- | --- | --- |
| One part of a holistic review | 59 (52.2) | 61 (54.0) |
| Tiebreaker between two equally qualified candidates | 45 (39.8) | 46 (40.7) |
| As a screening tool | 44 (38.9) | 52 (46.0) |
| Prioritizing wait list or wait list order | 31 (27.4) | 38 (33.6) |
| Interview invite to every applicant who signaled the program | 19 (16.8) | 17 (15.0) |
| As a discussion point during the interview | 16 (14.2) | 23 (20.4) |
| During rank order list discussion | 14 (12.4) | 17 (15.0) |
| To review all applications that were signaled regardless of initial filters within ERAS | 1 (0.9) | 0 (0) |
| To prioritize interview invitations for diverse applicants who signaled the program | 1 (0.9) | 1 (0.9) |
| To stratify the program’s hold list | 1 (0.9) | 0 (0) |
| As a filter for applications from parts of the country we recruit poorly from | 0 (0) | 1 (0.9) |
| *Notes:* ERAS = Electronic Residency Application Service | | |
